# Impact of Environmental Temperature on the Pathological Complete Response and Survival Outcomes of Breast Cancer: A NCDB and SEER study

**DOI:** 10.21203/rs.3.rs-2718368/v1

**Published:** 2023-03-28

**Authors:** Ashish Gupta, Kush Gupta, Arya Mariam Roy, Kristopher Attwood, Asha Gandhi, Stephen Edge, Kazuaki Takabe, Elizabeth Repasky, Song Yao, Shipra Gandhi

**Affiliations:** Roswell Park Comprehensive Cancer Center; University of Massachusetts Medical School – Baystate; Roswell Park Comprehensive Cancer Center; Roswell Park Comprehensive Cancer Center; SGT University; Roswell Park Comprehensive Cancer Center; Roswell Park Comprehensive Cancer Center; Roswell Park Comprehensive Cancer Center; Roswell Park Comprehensive Cancer Center; Roswell Park Comprehensive Cancer Center

**Keywords:** Breast Cancer, Environmental temperature, Cold stress, Pathologic complete response, Overall survival

## Abstract

**Background:**

Experimental evidence in tumor-bearing mouse models shows that exposure to cool, that is, sub-thermoneutral environmental temperature is associated with a higher tumor growth rate with an immunosuppressive tumor immune microenvironment than seen at thermoneutral temperatures. However, the translational significance of these findings in humans is unclear. We hypothesized that breast cancer patients living in warmer climates have higher odds of achieving pathologic complete response (pCR) and better survival outcomes than patients living in colder climates.

**Methods:**

A retrospective population-based analysis was conducted on Stage I-III breast cancer patients utilizing data from National Cancer Database (NCDB) from 2010–2018 with 892,092 patients and Surveillance, Epidemiology and End Results (SEER) from 1996–2017 with 270,496 patients. The average annual temperature (AAT) was calculated based on data from the National Centers for Environmental Information.

**Results:**

In the SEER cohort, patients residing at AAT ≥47.5°F had a 16% higher overall survival (OS) (HR 0.84, 95% CI 0.81–0.88, p <0.001) and 15% higher disease specific survival (DSS) (HR 0.85, 95% CI 0.80 – 0.90; p <0.001). Similarly, 4% higher OS (HR 0.96, 95% CI 0.95–0.97, p <0.001) and DSS (HR 0.96, 95% CI 0.94–0.97, p <0.001) was noted with every 5°F increment in AAT. In the NCDB cohort, patients in regions with AAT ≥ 60.9°F had 9% greater odds of achieving a pCR, odds ratio (OR 1.09, 95% CI 1.05, 1.13, p <0.001) and a 5% higher OS (HR 0.95, 95% CI 0.93 – 0.97, p<0.001).

**Conclusions:**

Higher environmental temperatures are associated with significantly better OS and DSS, as well as higher odds of achieving pCR in these patients. Future research is warranted to confirm this observation using large datasets, to elucidate the underlying mechanisms and investigate novel therapeutic strategies to minimize this geographic disparity in clinical outcomes.

## Background

The 2022 cancer statistics estimated 287,850 new cases of invasive breast cancer (BC) and > 43,250 related deaths in the United States (US) [1]. The survival outcomes of BC have undergone a paradigm shift in recent decades, with a reported 5-year survival of >85% in many high-income countries [2]. Nonetheless, there are global and regional disparities in BC survival outcomes, largely attributed to treatment availability, screening modalities, and the quality of care [3]. Recently, studies have identified geographical disparities in the survival trends of BC and efficacy of treatment modalities within the same country or healthcare system, which cannot be solely attributed to race, ethnicity or other patient-specific factors [4].

Several environmental, lifestyle, and metabolic factors promote tumorigenesis through genetic alterations, epigenetic changes, induction of pro-tumorigenic signaling pathways, and suppression of pro-apoptotic signaling [5–7]. Recent experimental evidence highlighted significant interactions between thermal stress, i.e., cool ambient housing temperature, and cancer progression, where stress responses and hormones were found to mediate molecular mechanisms linked to an immunosuppressive tumor microenvironment (TME) [8]. Kokolus et al. showed an association between sub-thermoneutral housing temperature (22°C) and tumor growth rate in tumor-bearing 4T1 murine models. Compared to sub-thermoneutral temperature, mice living under thermoneutral housing temperature (30 °C) showed significant reductions in tumor growth rate and metastasis; tumor exhibited a more favorable TME more anti-tumor CD8^+^T-cells and fewer pro-cancerous myeloid derived suppressor cells (MDSCs) and regulatory T-cells (Tregs)[9–11].

Epidemiological data suggests that countries with the coldest temperatures have the highest cancer incidence. Thus, a “cancer-cold” hypothesis was advocated by researchers, which proposes a significant association between low temperature and increased incidence of malignancy [12]. Similarly, a US population-based study found that people living in cold climate counties may have a higher cancer incidence, including BC, while controlling for confounding variables like ethnicity, age, or income [13], Sharma et al. reported that the incidence of 13 cancer types including breast cancer (out of the 16 studied cancers) was negatively correlated with the average annual temperature (AAT) [14].

Furthermore, Sharma et al also reported that patients with cancer living in countries with a higher AAT had a higher cancer-related mortality, compared to countries with a lower AAT. Many factors like lifestyle, social situation, healthcare system, reporting mechanisms etc. may affect the mortality rates across countries. In addition, temperature range within a country can be highly variable [15]. Nonetheless, the prognostic significance of environmental temperature on BC survival and pathological complete response (pCR) after neoadjuvant treatment, which is a surrogate marker for long-term outcomes, has not been studied yet. Thus, to the best of our knowledge this is the first study assessing the impact of environmental temperature on pCR, overall survival (OS) and disease specific survival (DSS) in stage I-III BC patients in the US. We hypothesized that BC patients living in warmer climates in the US have better OS, DSS and higher odds of achieving pCR compared to patients living in colder climates.

## Methods

### Study Design

A retrospective population-based analysis was conducted utilizing two national cancer databases, the National Cancer Database (NCDB) and the Surveillance, Epidemiology and End Results (SEER) Database. NCDB which is sponsored by the American College of Surgeons and American Cancer Society represents approximately 70% of newly diagnosed cancer cases in the US. It consists of patients who have received some element of their care at a facility accredited by the Commission on Cancer. The SEER database is from the National Cancer Institute (NCI), covering approximately 35% of the US population. Temperature data were obtained from the National Centers for Environmental Information (NCEI; https://www.ncei.noaa.gov/), which is a US government agency that provides access to environmental data, including average temperature data given by county, month, and year [16]. The NCEI collects temperature data from a variety of sources, including weather stations, buoys, ships, and satellites. The temperature data is collected using a variety of instruments and techniques, depending on the type of data source. For example, data from weather stations are typically collected using a thermometer. For satellites, temperature data are collected by measuring the infrared radiation emitted by the Earth’s surface and atmosphere. The temperature data is then calculated using algorithms that consider factors such as atmospheric conditions and surface emissivity[16].

Once the temperature data is collected, it is typically recorded at regular intervals, such as every hour or every 15 minutes, depending on the data source. The data is then transmitted to the NCEI, where it is processed, and quality controlled to ensure that it is accurate and consistent. The processed data is then made available to the public through various channels, including the NCEI website and other data portals.

For the SEER analysis, this data was utilized to obtain the monthly average temperature in a particular county at the year of the patient’s diagnosis and/or surgery, which was averaged to obtain the AAT. For the NCDB analysis, only regional location data is available based on the site of treatment. Thus, the average temperatures in a particular region were obtained and averaged to calculate the AAT.

Institutional Review Board approval was waived as the SEER, and NCDB cohorts are publicly available and de-identified.

### Study Population

We retrieved data of all adult (>18 years old) stage I-III BC patients diagnosed from 1996 to 2017 (SEER) and from 2010 to 2018 (NCDB). We identified the site of malignancy through the 3rd edition of the WHO International Classification of Diseases for Oncology. For NCDB, we excluded patients with missing information on pathological response (if they received neoadjuvant treatment) or survival data. For SEER we excluded patients who were missing information on staging, OS, and location information.

### Statistical Analysis

OS and DSS were analyzed using Kaplan-Meier methods in both the SEER and NCDB (OS only) datasets. Optimal thresholds for the AAT to identify poor vs better survival outcomes were obtained using the maximal log-rank approach. Response to neoadjuvant treatment (pCR) was analyzed in the NCDB dataset using frequencies and relative frequencies. Optimal thresholds for the AAT to discriminate between pCR and non-pCR were identified using the Youden’s index criterion.

Univariate associations between AAT and survival outcomes and were evaluated using cox and logistic regression models, respectively. Multivariable cox and logistic regression models were used to evaluate the association with AAT while accounting for additional covariates, which were identified using the backwards selection approach (alpha exit = 0.10). In SEER, the variables included were: age, race, ethnicity, marital status, rural or urban area, BC subtype, stage, grade of tumor, and type of treatment received. For NCDB, the variables additionally included were: Charlson/Deyo comorbidity score (CDCC), facility type, education and income. Subgroup analyses were performed according to receptor subtype. Hazard ratios (HR) or odds ratios (OR) corresponding to AAT were obtained from model estimates and presented with 95% confidence interval (CI).

All analyses were conducted in SAS v9.4 (Cary, NC) at a two-sided significance level of 0.05.

## Results

### Characteristics of Study Cohort

A total of 892,092 Stage I-III BC patients from NCDB and 270,496 from SEER database were analyzed. In the NCDB cohort, 99.1% were female, 83.3% were white, 69.3% were > 55 years old, 16.8% had a CDCC score of ^3^1. 73.6% had tumors that were hormone receptor positive (HR+)/human epidermal growth factor receptor 2 negative (HER2−), 12% were triple negative (TNBC)_, 10.1% were HR+/HER2+, and 4.3% were HR−/HER2+. Overall, 35.1% patients received chemotherapy 64.5% radiation and 94.3% surgery, and 9.2% patients received neoadjuvant chemotherapy; of them, 21.6% (17,691/81,926) achieved pCR. The AAT for all the regions in NCDB ranged from 45.2°Fahrenheit (°F) to 62.3°F with median temperature of 50.9°F. The details of the descriptive characteristics are shown in Table 1.

In the SEER Cohort, 99.3% were female, 77.9% were white, 62.6% were > 55 years old. Receptor status data was available for tumors of 32.5% patients. Among those with available data, 69.59% were HR+/HER2−, 13.29% TNBC, 11.97% HR+/HER2+, and 5.15% HR−/HER2+.43.4% received chemotherapy, 49.7% radiation and 94.2% surgery. The AAT for all the counties ranged from 33.6°F to 67.3°F with a median temperature of 57.4°F. The details of the descriptive characteristics are shown in Table 2.

### The association between OS and AAT

The median OS is not reached for the NCDB cohort at a median follow-up of 57.8 (0.0, 127.3) months. Based on the maximum log-rank approach, the optimal temperature threshold for the NCDB cohort was AAT ≥60.9°F.

With every 5°F increment in AAT at diagnosis, the unadjusted HR for OS was 1.05. After adjusting for potential confounders, there was a 2% higher OS with every 5°F increment in AAT at diagnosis (HR 0.98, 95% CI 0.97–0.99, p < 0.001). Likewise, for patients living at AAT ≥60.9°F the unadjusted HR was 1.07. After adjusting for potential confounders, there was a 5% higher OS (HR 0.95, 95% CI 0.93 – 0.97, p<0.001). Subtype-specific data for this association between AAT and OS are shown in Table 3. The summary of difference in characteristics for patients >60.9 vs <60.9 is shown in Supplementary Table 1 and this difference in characteristics i.e. confounders are likely responsible for change in direction of the result.

The median OS for the SEER cohort was 227 (95% CI 225 – 230) months. The unadjusted cox regression showed HR 0.99. After adjusting for potential confounders, there was a 4% higher OS (HR 0.96, 95% CI 0.95–.97, p < 0.001) and 4% higher DSS (0.96, 95% CI 0.94–0.97, p < 0.001) with every 5°F increment in AAT. Based on the maximum log-rank approach the optimal temperature threshold for the SEER cohort was AAT ≥ 47.5°F. Patients living at AAT ≥ 47.5°F the unadjusted HR was .91 for OS and .89 for DSS. After adjusting for potential confounders, there was a 16% higher OS (HR 0.84, 95% CI 0.81–0.88, p < 0.001) and 15% higher DSS (HR 0.85, 95% CI 0.80 – 0.90; p < 0.001) (Table 4).

### The association between pCR and AAT

Based on Youden’s index criterion approach the optimal temperature threshold for the NCDB cohort was AAT ≥ 60.9°F. In terms of pCR, when adjusting for covariates using the multivariate analysis and using Youden’s index criterion to determine optimal temperature cut-off, 10% higher odds were noted of achieving pCR with BC patients living in regions with AAT at surgery of ≥ 60.9°F (aOR 1.10, 95% CI 1.05, 1.16, p < 0.001) compared to AAT < 60.9°F. The odds were even higher for patients with TNBC (aOR 1.14, 95% CI 1.07, 1.23, p < 0.001) and HER2+HR− (aOR 1.14, 95% CI 1.03, 1.26, p = 0.015). No significant difference was observed with every 5°F increase in temperature and pCR (aOR 1.00, 95% CI 0.98, 1.02, p = 0.955).

## Discussion

Ambient temperature is an important driver of human health, with well-established associations between ambient temperature changes and disease burden [17], mortality [18], and morbidity [19]. Preclinical and epidemiologic data has suggested an association between cancer progression and climate. Our analysis demonstrates that higher AAT was an independent predictor of improvement in OS, DSS, and pCR in stage I-III BC patients. We found that patients living in areas with an AAT ≥60.9°F had 10% higher odds of achieving a pCR. In the SEER cohort, patients living at AAT ≥ 47.5°F had a 16% improvement in OS and 15% improvement in DSS, whereas, in the NCDB cohort, a 5% improvement in OS was noted at optimal temperature cutoff of AAT ≥60.9°F. With every 5°F increment in AAT, a 4% improvement in OS and DSS was noted in the SEER cohort, whereas a 2% improvement in OS was noted in the NCDB cohort. The differences between SEER and NCBD cohorts may be attributed to the availability of more specific county-level data with the SEER cohort vs regional data with NCDB. The results also indicate that the impact of AAT on survival was more notable when data were grouped according to optimal temperature cut-off values. While the exact causes of such findings are unclear, we speculate that the greater impact at more extremes of temperature may indicate that the effect of temperature may not be linear and at lower or higher temperatures, a tipping point may be reached accounting for these differences.

The findings that a warmer climate is associated with favorable survival outcomes and a higher chance of achieving pCR in stage I-III BC patients is in line with our recent publication showing a trend towards worse survival in patients whose tumors have high thermogenesis score (which is shown to be increased in cold weather) [20]. We showed that high thermogenesis TNBC i.e., tumors under thermal stress, was associated with an immunosuppressive TME and a significantly worse DSS[20].

While the exact mechanisms and pathways underpinning the negative impact of cold climate on oncological outcomes have not been fully elucidated yet, we propose several explanations for these findings. We hypothesize that impaired anti-tumor immune responses in the TME under cold stress contribute to the observed worse outcomes amongst patients living in a colder climate. As previously mentioned, sub-thermoneutral temperatures were associated with impaired immune response in the TME. Housing mice under these temperatures resulted in a lower number of functional anti-tumor CD8^+^ T and overexpression of MDSCs [21], a potent suppressor of anti-tumor immune responses and CD8+ T proliferation [21,22]. Besides, Bucsek et al. found Tregs and programmed death receptor-1 (PD-1) overexpression in mice exposed to cold stress and showed that mice housed at thermoneutral temperature of 30°C exhibited an anti-tumor immune microenvironment with higher number of CD8+ T cells and lower number of MDSCs and T-regs than mice that were kept at the sub-thermoneutral temperature of 22°C [23].

pCR is a commonly utilized surrogate marker for long-term survival benefits in neoadjuvant BC clinical trials. Previous reports have demonstrated that pCR is significantly associated with prolonged event-free survival and OS, with a stronger association amongst patients with more aggressive BC subtypes (TNBC and HER2+) [24,25]. Thus, regulatory authorities have supported the use of pCR in BC trials as a surrogate endpoint for long-term survival outcomes for accelerated approval of therapies [26,27]. This study investigated the impact of pCR, alongside the traditional survival outcomes, owing to its well-established prognostic utility.

Our study findings that validate the association between temperature and outcomes in BC have clinical and translational implications. Our study validates the results of pre-clinical data noting an association between tumor behavior and temperature. It was also shown that housing temperature alters mouse biology and anti-tumor immune responses and sensitivity of tumors to therapeutic interventions [8]. Therefore, the housing temperature of cancer models should be considered during the assessment of therapeutic interventions, alongside the consideration of climate as a possible confounder during the clinical assessment of novel therapies. Building on this knowledge of immunosuppression in cold temperatures, targeted interventions for neural thermoreceptive pathways are proposed for improving clinical outcomes amongst patients living in a colder climate. In a phase I clinical trial, adding a non-selective β-blocker to pembrolizumab, an immune checkpoint inhibitor, led to an impressive objective response rate of 78% in patients with metastatic melanoma [28]. In BC models, non-selective β-blocker reversed the effects of cold stress on tumor growth and spread [29]. In a phase II clinical trial, 60 BC patients were randomized to receive propranolol or placebo one week preoperatively. The results showed that propranolol improved the anti-tumor immune response and reduced biomarkers associated with metastatic potential [30].

The drivers of tumorigenesis and alteration in the TME in those who live in colder environments can be analyzed in detail using metabolomics which involves the measurements of various small molecule metabolites including signaling mediators, nutrients, proteins in the blood, and the metabolic products of these molecules in the body fluids [31]. This would help us to develop personalized treatment options for those with aggressive malignancies who live in colder environments. As cold therapy for alopecia [32] and cryotherapy to prevent chemotherapy-induced neuropathy is gaining momentum [33], the clinical outcomes of patients who undergo these treatments are worth examining to scrutinize possible impact on their clinical outcomes with these novel interventions. In recent years, treatments involving hyperthermia have been utilized with success in multiple cancers. Hyperthermia when combined with chemotherapy and immunotherapy has shown promising results in gynecological malignancies treatments including Hyperthermic Intraperitoneal Chemotherapy (HIPEC) [34] and has increased therapeutic effectiveness in melanoma when combined with radiation therapy [35]. Given the finding from our study that shows that BC patients who live in warm climatic regions have a better prognosis, we could potentially utilize the benefit of hyperthermia treatments in BC in the future.

Our study has the strengths of a large sample size and representation of the US population. Also, we used two large national databases to investigate our hypothesis, and both showed similar findings. These findings were in line with our preclinical studies showing a significant association between ambient temperature and TME. Nonetheless, the study has several limitations. First, the analysis is based on retrospective data, which limits the control of the outcomes. BC subtype data were missing in a considerable proportion of the SEER cohort. Data regarding some socioeconomic variables, such as education and income, were not available in the SEER database, thus was not able to be adjusted for in this analysis. However, data on these variables was available in the NCDB analyses and was adjusted for. Despite these differences in the two databases, the clinical outcomes were similar. In terms of AAT, the NCDB provides regional location data; hence, we could not account for the variation in temperature across each region. Patients may have changed their residence during the course of surgery and follow-up, which can influence our results and therefore a time-dependent analysis might not be as informative. Since this is an ecological study examining the relationship between clinical outcome and temperature exposure at a population level, individual level data (e.g. air-conditioning, personal and lifestyle factors) was not available. Unlike mice, humans can mitigate cold stress by changing their environment. For example, some people are more exposed to the effects of ambient temperature, air pollution, light e.g., construction workers, vs. others who spend the majority of their time in a temperature-controlled setting, e.g., desk workers, and we were not able to account for this disparity in our analysis. The limitation of SEER was that it does not provide pCR data. Therefore, pCR analysis was done only with the NCDB. Another limitation of our study is that there is overlap among patients whose data is collected both, using SEER and NCDB databases. However, we decided to use both databases, because SEER provides us data on DFS while NCDB provides us data on pCR.

## Conclusions

In conclusion, higher environmental temperatures are associated with significantly better oncological outcomes in stage I-III BC patients. Considering the limitations of the current study using SEER/NCDB databases, future research is warranted to confirm this observation using center-specific and/or prospective studies which would provide more detailed data about monthly temperature and patients’ whereabouts to consider a time dependent study model, and help us dissect data to understand the underlying mechanisms and develop strategies to address this disparity in clinical outcomes.

## Figures and Tables

**Table 1:** Characteristics of NCDB Cohort (n =892,097patients)

Overall	N	892,097
AAT (°F)	Range 45.2^o^F-62.3^o^F median 50.9^o^F	
Age at Diagnosis (in years)	Mean/Std/N	61.8/12.0/892097
	Median/Min/Max	61.0/40.0/90.0
Age (in years)	< 45	63,050 (7.1%)
	45–55	210,411 (23.6%)
	55–65	253,469 (28.4%)
	>= 65	365,167 (40.9%)
Sex	Male	7,906 (0.9%)
	Female	884,191 (99.1%)
Race	White	736320 (82.5%)
	Black	102551 (11.5%)
	Other	45090 (5.1%)
	Missing	8136 (0.9%)
Ethnicity	Non-Hispanic	815142 (91.4%)
	Hispanic	50969 (5.7%)
	Missing	25986 (2.9%)
Income	<$38,000	115013 (12.9%)
	$38,000-$47,999	163205 (18.3%)
	$48,000-$62,999	208782 (23.4%)
	>=$63,000	295551 (33.1%)
	Missing	109546 (12.3%)
Education	>=21%	117809 (13.2%)
	13.0–20.9%	184233 (20.7%)
	7.0–12.9%	256620 (28.8%)
	< 7.0%	224160 (25.1%)
	Missing	109275 (12.2%)
Facility Type	Community CP	67513 (7.6%)
	Comprehensive CCP	372478 (41.8%)
	Academic/Research	260037 (29.1%)
	Integrated Network CP	192069 (21.5%)
Urban/Rural	Metro	778224 (87.2%)
	Urban	101010 (11.3%)
	Rural	12863 (1.4%)
CDCC	0	742285 (83.2%)
	1	115534 (13.0%)
	2	24241 (2.7%)
	>=3	10037 (1.1%)
Grade	Well differentiated, differentiated, NOS	199149 (22.3%)
	Moderately differentiated,	380238 (42.6%)
	Poorly differentiated	261575 (29.3%)
	Undifferentiated, anaplastic	1499 (0.2%)
	Missing	49636 (5.6%)
Tumor Size (mm)	Mean/Std/N	22.4/28.0/657915
	Median/Min/Max	16.0/0.0/989.0
Clin Stage	1	541425 (60.7%)
	2	284209 (31.9%)
	3	66463 (7.5%)
Path Stage	0	11907 (1.3%)
	1	449161 (50.3%)
	2	251454 (28.2%)
	3	71351 (8.0%)
	4	1619 (0.2%)
	Missing	106605 (11.9%)
Sub-Type	TNBC	101295 (11.4%)
	HR+/HER2−	621688 (69.7%)
	HR+/HER2+	85187 (9.5%)
	HR−/HER+	36045 (4.0%)
	Missing	47882 (5.4%)
Laterality	Origin of primary is right	439957 (49.3%)
	Origin of primary is left	451942 (50.7%)
	Only one side involved, right or left or	198 (0.02%)
Surgery	No	50457 (5.7%)
	Yes	841640 (94.3%)
Radiation	None	318425 (35.7%)
	Neoadjuvant	2413 (0.3%)
	Adjuvant	551013 (61.8%)
	Both	331 (0.03%)
	Missing	19915 (2.2%)
Chemotherapy	None	521788 (58.5%)
	Neodjuvant	90637 (10.2%)
	Adjuvant	222096 (24.9%)
	Both	4513 (0.5%)
	Missing	53063 (5.9%)
Immunotherapy	No	819957 (91.9%)
	Yes	69293 (7.8%)
	Missing	2847 (0.3%)
Hormone Therapy	No	250062 (28.0%)
	Yes	621582 (69.7%)
	Missing	20453 (2.3%)
pCR	No pCR	64235 (7.2%)
	pCR	17691 (2.0%)
	Missing	8711 (1.0%)

AAT: Average annual temperature; ^o^F: Fahrenheit; Std: Standard deviation; NOS: Not otherwise specified; TNBC: Triple-negative breast cancer; HR: Hormonal receptor; HER2: human epidermal growth factor receptor 2; pCR: Pathological complete response; CDCC- Charlson Deyo Comorbidity Score

**Table 2:** Characteristics of SEER Cohort (n =270,496 patients)

Overall		I/II/III
		270,496
**AAT (°F)**	AAT 33.6^o^F-67.3^o^F	median 57.4^o^F
**Age (years)**	Mean/SD/N	59.91/13.74/270,492
**Age (years)**	< 45	36,828 (13.61%)
	45–55	64,971 (24.02%)
	55–65	68,048 (25.16%)
	≥ 65	100,645 (37.21 %)
**Race**	White	211,589 (78.22%)
	Black	39,251 (14.51%)
	Other	19,656 (7.27%)
**Ethnicity**	Not Hispanic	252,853 (93.48%)
	Hispanic	17,643 (6.52%)
**Sex**	Female	268,585 (99.29%)
	Male	1,911 (.71%)
**Married**	Married	150,376 55.59%)
	Single	120,120 44.41 %)
**Urban**	Rural	32,660 (12.07%)
	Urban	237,836 (87.93%)
**Grade**	I/II	158,213 (58.49%)
	III/IV	94,241 (34.84%)
	Unknown	18,042 (6.67%)
**Laterality**	Left	137,214 (50.73%)
	Right	133,282 (49.27%)
**Breast cancer subtypes**	HR−/HER2+	4,518 (5.15%)
	HR+/HER2−	61,080 (69.59%)
	HR+/HER2+	10,506 (11.97%)
	Triple Negative	11,663 (13.29%)
	Unknown	182,729 (67.55%)
**Chemotherapy**	Yes	115,901 (42.85%)
	None/Unknown	154,595 (57.15%)
**Radiation**	Yes	138,875 (51.34%)
	No	129,382 (47.83%)
	Unknown	2,239 (0.83%)
**Surgery**	Yes	262,255 (96.95%)
	No	7,894 (2.92%)
	Unknown	347 (.13%)

AAT: Average annual temperature; °F: Fahrenheit; Std: Standard deviation; NOS: Not otherwise specified; TNBC: Triple-negative breast cancer; HR: Hormonal receptor; HER2: human epidermal growth factor receptor 2; pCR: Pathological complete response

**Table 3:** Univariate and Multivariate Analysis of AAT as predictor of OS and pCR in NCDB Cohorts

Variable		OS			Variables		pCR		
		Unadjusted HR (95% CI	Adjusted HR (95% CI)	P-value			Unadjusted OR (95% CI	Adjusted OR (95% CI)	P-value
Every 5-degree increment		1.05 (1.04, 1.06)	0.98 (0.97, 0.99)	<0.001	Every 5-degree increment		0.995 (0.98–1.01)	1.00 (0.98 – 1.02)	0.955
AAT at diagnosis	≤60.9	Ref	Ref	<0.001	AAT at surgery	≤60.9	Ref		
	>60.9	1.07 (1.06,1.09)	0.95 (0.93, 0.97)			>60.9	1.09 (1.05, 1.13)	1.10 (1.05 −1.16)	<0.001
Subtypes (Ref AAT ≤ 60.9)	TNBC	1.03 (0.99, 1.07)	0.95 (0.91,0.99)	0.013	Subtypes (Ref AAT ≤ 60.9)	TNBC	1.10 (1.031.17)	1.14 (1.07, 1.23)	<0.001
	HER2+HR−	1.09 (1.01, 1.17)	0.94 (0.86, 1.03)	0.198		HER2+HR−	1.02 (0.941.11)	1.14 (1.03, 1.26)	0.015
	HR+HER2−	1.05 (1.03, 1.08)	0.94 (0.92, 0.97)	<0.001		HR+HER2−	1.09 (1.001.20)	1.03 (0.92, 1.15)	0.583
	HR+HER2+	1.14 (1.07, 1.20)	1.02 (0.95, 1.10)	0.565		HR+HER2+	1.02 (0.901.16)	1.07 (0.92, 1.24)	0.379

OS; Overall survival; pCR: Pathological complete response; AAT: Average annual temperature; TNBC: Triple-negative breast cancer; HR: Hormonal receptor; HER2: human epidermal growth factor receptor 2; HR: Hazard ratio

**Table 4:** Univariate and Multivariate Analysis of AAT as predictor of OS and DSS in SEER Cohort

Variable		OS			DSS		
		Unadjusted HR (95% CI	Adjusted HR (95% CI)	P-value	Unadjusted HR (95% CI	Adjusted HR (95% CI)	P-value
Every 5-degree incremenl		0.99 (0.98 – 1.0)	0.96 (0.95, 0.97)	<0.001	0.98 (0.97 – 0.99)	0.96 (0.94, 0.97)	<0.001
AAT at diagnosis	≤47.5	Ref	Ref	<0.001	Ref	Ref	
	>47.5	0.91 (0.89–0.92)	0.84 (0.81, 0.88)		0.89	0.85 (0.80, 0.90)	<0.001
Subtypes (Ref AAT ≤ 47.5)	TNBC	0.90 (0.82, 0.98)	0.90 (0.82, 0.99)	0.025	0.90 (0.81, 1.00)	0.92 (0.83, 1.02)	0.122
	HER2+HR−	0.76 (0.63, 0.91)	0.82 (0.68, 0.99)	0.037	0.72 (0.58, 0.89)	0.79 (0.64, 0.99)	0.036
	HR+HER2−	0.82 (0.77, 0.87)	0.84 (0.79, 0.89)	<0.001	0.82 (0.74, 0.90)	0.84 (0.77, 0.93)	<0.001
	HR+HER2+	0.74 (0.64, 0.85)	0.73 (0.63, 0.85)	<0.001	0.64 (0.53, 0.77)	0.65 (0.54, 0.79)	<0.001

NE: Not estimated; OS; Overall survival; DSS: Disease-specific survival; AAT: Average annual temperature; HR: Hazard ratio; TNBC: Triple-negative breast cancer; HR: Hormonal receptor; HER2: human epidermal growth factor receptor 2

## Data Availability

The datasets generated and/or analyzed during the current study are available publicly from the American College of Surgeons and the Surveillance, Epidemiology, and End Results Program.

## Abbreviations

AAT: average annual temperature
BC: breast cancer
C: Celsius
CDCC: Charlson/Deyo comorbidity score
CI: Confidence Interval
DSS: disease specific survival
F: Fahrenheit
HER2: human epidermal growth factor receptor 2
HIPEC: Hyperthermic Intraperitoneal Chemotherapy
HR: hazard ratio
HR: hormone receptor
MDSCs: myeloid derived suppressor cells
NCDB: National Cancer Database
NCEI: National Centers for Environmental Information
NCI: National Cancer Institute
OR: odds ratio
OS: overall survival
pCR: pathologic complete response
PD-1: programmed death receptor-1
SEER: Surveillance, Epidemiology and End Results
T-regs: regulatory T-cells
TME: tumor microenvironment
TNBC: triple negative breast cancer
US: United States
